# Loading of Beclomethasone in Liposomes and Hyalurosomes Improved with Mucin as Effective Approach to Counteract the Oxidative Stress Generated by Cigarette Smoke Extract

**DOI:** 10.3390/nano11040850

**Published:** 2021-03-26

**Authors:** Maria Letizia Manca, Maria Ferraro, Elisabetta Pace, Serena Di Vincenzo, Donatella Valenti, Xavier Fernàndez-Busquets, Catalina Anisoara Peptu, Maria Manconi

**Affiliations:** 1 Department of Life and Environmental Sciences, University of Cagliari, Via Ospedale 72, 09124 Cagliari, Italy; dvalenti@unica.it (D.V.); manconi@unica.it (M.M.); 2 Istituto per la Ricerca e l’Innovazione Biomedica (IRIB), CNR, Via Ugo La Malfa 153, 90146 Palermo, Italy; maria.ferraro@irib.cnr.it (M.F.); elisabetta.pace@irib.cnr.it (E.P.); serena.divincenzo@irib.cnr.ir (S.D.V.); 3 Institute for Bioengineering of Catalonia (IBEC), The Barcelona Institute of Science and Technology, Baldiri Reixac 10-12, 08028 Barcelona, Spain; xfernandez_busquets@ub.edu; 4 Barcelona Institute for Global Health (ISGlobal, Hospital Clínic-Universitat de Barcelona), Rosselló 149-153, 08036 Barcelona, Spain; 5 Nanoscience and Nanotechnology Institute (IN2UB), University of Barcelona, Martí i Franquès 1, 08028 Barcelona, Spain; 6 Department of Natural and Synthetic Polymers, Faculty of Chemical Engineering and Environmental Protection, “Gheorghe Asachi” Technical University of Iasi, Bulevardul Profesor Dimitrie Mangeron 67, 700050 Iasi, Romania; catipeptu@yahoo.co.uk

**Keywords:** beclomethasone, phospholipid vesicles, mucin, 16HBE cells, oxidative stress, pulmonary delivery, cigarette smoke extract

## Abstract

In this work beclomethasone dipropionate was loaded into liposomes and hyalurosomes modified with mucin to improve the ability of the payload to counteract the oxidative stress and involved damages caused by cigarette smoke in the airway. The vesicles were prepared by dispersing all components in the appropriate vehicle and sonicating them, thus avoiding the use of organic solvents. Unilamellar and bilamellar vesicles small in size (~117 nm), homogeneously dispersed (polydispersity index lower than 0.22) and negatively charged (~−11 mV), were obtained. Moreover, these vesicle dispersions were stable for five months at room temperature (~25 °C). In vitro studies performed using the Next Generation Impactor confirmed the suitability of the formulations to be nebulized as they were capable of reaching the last stages of the impactor that mimic the deeper airways, thus improving the deposition of beclomethasone in the target site. Further, biocompatibility studies performed by using 16HBE bronchial epithelial cells confirmed the high biocompatibility and safety of all the vesicles. Among the tested formulations, only mucin-hyalurosomes were capable of effectively counteracting the production of reactive oxygen species (ROS) induced by cigarette smoke extract, suggesting that this formulation may represent a promising tool to reduce the damaging effects of cigarette smoke in the lung tissues, thus reducing the pathogenesis of cigarette smoke-associated diseases such as chronic obstructive pulmonary disease, emphysema, and cancer.

## 1. Introduction

Exposure to cigarette smoke causes severe damage, especially at lung level, by means of the release of toxic mediators, including proteolytic enzymes and reactive oxygen species [1]. The chronic presence of these toxic molecules in the lungs has been linked to a variety of chronic disorders such as chronic obstructive pulmonary disease, emphysema and cancer, which are considered the main causes of morbidity and mortality in modern society [2]. Indeed, smoking is a major contributor to lung cancer and chronic obstructive pulmonary disease [3]. The latter is considered the most important nonmalignant lung disease caused by cigarette smoke [4]. This progressive health problem causes lung inflammation, the gradual destruction of the airways and lung parenchyma, and often degenerates in emphysema and can favor the development of lung cancer [5].

Different strategies have been carried out to prevent or reduce the damaging effects of cigarette smoke in airways. The use of effective active substances is considered one of the most efficient and safe approaches to prevent lung diseases associated with oxidative stress [6]. The most successful therapies for the treatment of these diseases involve the use of glucocorticoids, which are potent anti-inflammatory drugs capable of counteracting the activity of the different cells involved in the inflammatory processes. Among all, beclomethasone dipropionate has been considered the first choice for the treatment of these diseases and is largely used in therapy. It is a structural analogue of cortisol, the main glucocorticoid hormone produced by the adrenal cortex, and acts by stimulating the expression of anti-inflammatory proteins and by inhibiting the activity of proinflammatory transcription factors [7,8,9,10]. Unfortunately, the constant use of systemic beclomethasone dipropionate can address important side effects [11]. Nowadays, inhalation is the preferred route for its administration as it is easy, well accepted by patients and ensures a local delivery to the deep lungs, avoiding systemic distribution and reducing side effects. An additional advantage connected with pulmonary delivery is the large area of absorption together with a highly permeable mucosa. Considering the low adverse effects associated with aerosol therapy, beclomethasone dipropionate may be also proposed to prevent the severe damages caused by cigarette smoke in the lungs. However, by the inhalation route, it is important to reach the deeper airways by an adequate drug delivery system, which can improve therapeutic efficacy and decrease undesirable side effects of drug [12]. In our previous studies, phospholipid vesicles formulated ad hoc disclosed optimal performances as lung delivery systems for different molecules [13,14]. The high versatility of these vesicles permits us to modify their structure, surface charge and adhesiveness, adapting the delivery capabilities to the target site.

Considering these promising results, in the present study, liposomes and hyalurosomes were selected as vesicles to load and deliver beclomethasone dipropionate. Hyalurosomes were chosen because sodium hyaluronate may improve the vesicle bioadhesiveness and act as a targeting moiety to the epithelial cells expressing cluster determinant 44 (CD44) [15,16]. Furthermore, the important role of sodium hyaluronate in counteracting pulmonary inflammation has been reported [17,18,19]. Additionally, the vesicles were improved by adding mucin, a high molecular weight glycoprotein present in different mucous secretions [20], which can facilitate the adhesion to and the passage of the vesicles through the mucous membranes of the airways. In addition, previous studies have shown that mucins provide significant protection against oxidants [4]. Vesicles were characterized by measuring mean diameter, size distribution, surface charge, entrapment efficiency and stability on storage. The aptitude of the vesicles to be nebulized has been evaluated in vitro by using the Next Generation Impactor (NGI). Finally, the biocompatibility and the ability of vesicles to reduce the oxidative stress induced by cigarette smoke were tested using 16HBE bronchial epithelial cell line.

## 2. Materials and Methods

### 2.1. Materials

Lipoid S75 (S75), a mixture of soybean phospholipids (~70% phosphatidylcholine, 9% phosphatidylethanolamine and 3% lysophosphatidylcholine), triglycerides and fatty acids, was kindly provided by AVG S.r.l. (Garbagnate Milanese, Milan, Italy), local supplier for Lipoid GmbH (Ludwigshafen, Germany). Sodium hyaluronate was purchased from DSM Nutritional Products AG Branch Pentapharm (Rheinfelden, Switzerland). Beclomethasone dipropionate, mucin and all the other reagents were of analytical grade and were purchased from Sigma-Aldrich (Milan, Italy). Reagents and plastics for cell culture were purchased from Life Technologies Europe (Monza, Italy).

### 2.2. Preparation of Vesicles

The vesicles were obtained by means of direct sonication, avoiding the use of organic solvents. S75 (60 mg/mL) and beclomethasone (1 mg/mL) were weighed in glass tubes and hydrated with phosphate buffered saline (PBS) to obtain liposomes. Mucin-liposomes were prepared by hydrating both phospholipid and drug with a solution of mucin (0.05%) in PBS. Hyalurosomes and mucin-hyalurosomes were obtained by weighing phospholipid, drug and sodium hyaluronate (5 mg/mL) in glass tubes and hydrating them with PBS or with a solution of mucin (0.05%) in PBS, respectively.

The obtained dispersions were sonicated (25 + 25 cycles 5 on 2 off, interspersed with 5 min pauses to allow the cooling of the sample) by using a Soniprep 150 sonicator (MSE Crowley, London, United Kingdom) in order to obtain small and homogeneous vesicles [21]. The amount (mg/mL) of components used to prepare the vesicles is reported in Table 1.

### 2.3. Characterization of Vesicles

Formation and morphology of vesicles were evaluated by cryogenic transmission electron microscopy (cryo-TEM). Sample (5 µL) was applied on a grid Lacey carbon film (Electron Microscopy Science, Hatfield, PA, USA). The grid was mounted on an automatic plunge freezing apparatus (Vitrobot FEI, Eindhoven, The Netherlands) to control humidity and temperature, immersed in liquid ethane, fast cooled from outside by liquid nitrogen, avoiding the formation of ice crystals. Observation was made at −170 °C in a Tecnai F20 microscope (FEI, Eindhoven, The Netherlands) operating at 200 kV, equipped with a cryo-specimen holder Gatan 626 (Warrendale, PA, USA). Digital images were recorded with an Eagle FEI camera, 4098 × 4098 pixels. Magnification between 20,000–30,000× and a defocus range of 2–3 μm was used [22].

Average diameter and polydispersity index of each sample was evaluated by means of photon correlation spectroscopy by using a Zetasizer Nano (Malvern Instruments, Worcestershire, UK). The zeta potential was measured by means of M3-PALS method (phase analysis light scattering) by using the Zetasizer Nano. Before the analysis, the samples were diluted with PBS (1:100).

A stability study was performed by monitoring the size and size distribution of the vesicles stored at room temperature (25 ± 1 °C) for 5 months.

### 2.4. Determination of Entrapment Efficiency of Vesicles

To evaluate the amount of beclomethasone dipropionate loaded into the vesicles, dispersions (2 mL) were purified by dialysing them (Spectra/Por^®^ 172 membranes: 12–14 kDa 173 MW cut-off, 3 nm pore size; Spectrum Laboratories Inc., DG Breda, Netherlands) against PBS (2 litres) for 2 h at room temperature (~25 °C). The medium was replaced after 1 h to improve the solubilization and the removal of the non-entrapped drug. The entrapment efficiency (E) was calculated as percentage of the drug found in the vesicle dispersions after purification with respect to the amount detected in the vesicles after their preparation. The amount of beclomethasone recovered in unpurified and purified vesicles was measured by HPLC after disruption of vesicles with methanol (dilution 1:100). The drug absorbance was measured at 240 nm, by using a chromatograph Alliance 2690 (Waters, Italy) equipped with a photodiode detector and a computer integrating apparatus (EmpowerTM 3) [23]. The column XSelect C18 (3.5 μm, 4.6 × 150 μm^2^) was used for the analysis. A mixture of water, acetic acid, acetonitrile (30.97: 0.03: 69 *v*/*v*) delivered at a flow rate of 1 mL/min was used as the mobile phase. Under these chromatographic conditions three standard solutions of beclomethasone were prepared and used to build a calibration curve [24].

### 2.5. Nebulization of Formulations and Aerodynamic Behaviour

The in vitro deposition of dispersions was evaluated by using the Next Generation Impactor (Eur. Ph 7.2, Copley Scientific Ltd., Nottingham, UK) and the PariSX^®^ air jet nebulizer connected to a ParyBoySX^®^ compressor [14,22,25,26]. Vesicle dispersions (3 mL) were placed in the jet nebulizer and aerosolized to dryness directly into the throat of the impactor. At the end of the experiment, the sample deposited into the different stages of the impactor was recovered with methanol and drug content was quantified by HPLC as reported above (Section 2.4). Deposition performances were evaluated calculating the percentage of total mass output (TMO), the fine particle dose (FPD), and the fine particle fraction (FPF) [25]. Mass median aerodynamic diameter (MMAD) and geometric standard deviation (GSD) values were calculated avoiding the inclusion of the mass deposited in the induction port [22,25]. The cumulative amount of particles with a diameter lower than the stated size of each stage was plotted as a percentage of recovered drug versus the cut-off diameter, and the MMAD of the particles was extrapolated from the graph [14].

### 2.6. Culture of Bronchial Epithelial Cells

16HBE is an immortalized cell line of human bronchial epithelial cells, which were chosen because their similarity in morphology and functions to the normal airway epithelial cells [27]. The cells were cultured in a humidified atmosphere at 5% CO_2_ and 37 °C by using Eagle’s minimum essential medium (MEM; Gibco-Thermo Fisher Scientific Waltham, MA, USA), enriched with 10% heat-inactivated fetal bovine serum (FBS), 1% mixture of nonessential amino-acids, 2 mM L-glutamine and 0.5% gentamicin as previously described [28].

### 2.7. Cell Viability Assay

The biocompatibility of vesicles was evaluated by means of the CellTiter 96^®^ Aqueous One Solution Cell Proliferation Assay (Promega, Madison, WI, USA), a colorimetric method capable of determining the number of viable cells by using MTS [3-(4,5-dimethylthiazol-2-yl)-5-(3-carboxymethox-yphenyl)-2-(4-sulfopheyl) 2H-tetrazolium] [29].

Cells (1 × 10^4^ cells/well) were grown in 96-well plates and treated with beclomethasone dipropionate in dispersion or loaded in vesicles (final concentration of beclomethasone dipropionate 10^−6^ M, 10^−8^ M, 10^−10^ M, 10^−12^ M). At the end of the treatment, 20 μL of CellTiter 96^®^ AQueous One Solution reagent was added to each well and the plates were incubated for 20 min at 37 °C and 5% of CO_2_. The absorbance was measured by using a microplate reader at 490 nm (Microplate reader wallacVictor2 1420 Multilabel Counter, Perkin Elmer, Turku, Finland). Results were expressed as percentage of viability compared with untreated cells (100% viability) [30].

### 2.8. Preparation of Cigarette Smoke Extract and Treatment of Cells

Kentucky 3R4F research-reference cigarettes (The Tobacco Research Institute, University of Kentucky) without filter were used. Cigarette smoke extract was prepared using a peristaltic pump Watson-Marlow 323 E/D (Rotterdam, The Netherlands). Briefly, each cigarette was smoked for 5 min, and two cigarettes were used to generate 20 mL of cigarette smoke extract solution in PBS. The resulting solution was filtered through a 0.22 μm pore filter to remove bacteria and large particles and used within 30 min of preparation. This solution was considered to be 100% cigarette smoke extract and, in each well, it was opportunely diluted in the medium up to 20%. Two cigarettes were smoked in 20 mL of PBS solution aiming at creating the smoke solution (cigarette smoke extract). To standardize this procedure, for each preparation we measured the osmotic dehydration of smoke solution (i.e., cigarette smoke extract absorbance) by measuring the absorbance at 320 nm. The pattern of absorbance, among different batches, showed very few differences, and the mean value of the different batches was 1.37 ± 0.16, as previously described [31]. The presence of contaminating lipopolysaccharide in undiluted cigarette smoke extract solution was assessed by a commercially available kit (Cambrex Corporation, East Rutherfort, NJ, USA) and was below the detection limit of 0.1 EU/mL.

Cells (1 × 10^4^ cells/well) were grown in 12-well plate, stressed with cigarette smoke extract 20% and treated for 24 h with beclomethasone dipropionate (10^−9^ M) in dispersion or loaded in vesicles. Each experiment was performed in triplicate.

### 2.9. Analysis of Intracellular Reactive Oxygen Species (ROS)

Intracellular ROS were measured by following the conversion of the nonfluorescent dichlorodihydrofluorescein diacetate (Sigma Aldrich, Milan, Italy) into a highly fluorescent compound, dichlorofluorescein, by monitoring the cellular esterase activity in the presence of peroxides as previously described [32]. The ROS generation was assessed by inducing the uptake of 1 μM nonfluorescent dichlorodihydrofluorescein diacetate and incubating for 10 min at room temperature in the dark, followed by flow cytometric analysis (CytoFLEX BeckmanCoulter, Brea, CA, USA). The results are expressed as percentage of ROS positive cells, stressed with cigarette smoke extract.

### 2.10. Statistical Data Analysis

The results were expressed as the mean values ± standard deviations. Statistically significant differences were determined using the analysis of variance, and the Student’s *t* test. The minimum significance level chosen was *p* < 0.05.

## 3. Results

### 3.1. Characterization of Vesicles

**Figure 1:**
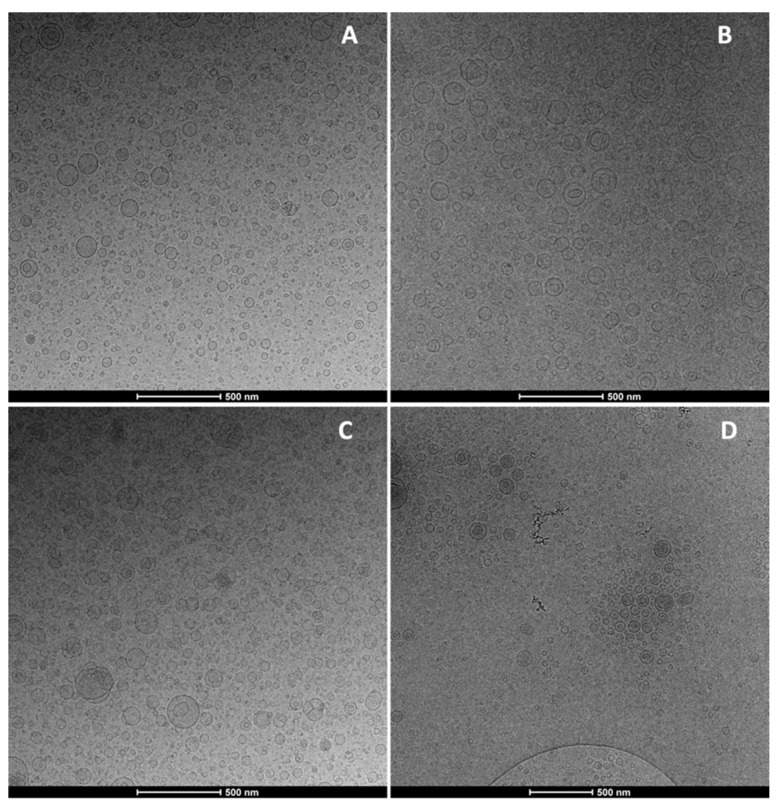
Representative Cryo-TEM images of beclomethasone dipropionate loaded liposomes (**A**), mucin-liposomes (**B**), hyalurosomes (**C**) and mucin-hyalurosomes (**D**).

The effective formation of the vesicles and their structure and morphology were observed by Cryo-TEM. The images confirmed the presence of vesicles small in size and regularly shaped. Liposomes were mostly unilamellar and the addition of mucin or sodium hyaluronate or their combination did not significantly change the morphology of the vesicles, which remained regularly shaped and mostly oligolamellar (Figure 1).

The mean diameter of the vesicles was measured by dynamic laser light scattering (Table 2). Liposomes and mucin-liposomes were the smallest vesicles (~105 nm, *p* > 0.05 between the values of the two samples), while the polydispersity index was always lower than 0.22, demonstrating a good homogeneity of the system. Hyalurosomes and mucin-hyalurosomes were slightly larger than the corresponding liposomes (~129 nm, *p* > 0.05 between the values of the two samples). All the prepared vesicles were negatively charged (~−11 mV) and this parameter was not affected by the presence of polymers.

All the vesicles were able to incorporate a high amount of beclomethasone, as the entrapment efficiency (E) was always higher than 81% irrespective of the vesicle composition (*p* > 0.05).

### 3.2. Stability Studies

**Figure 2:**
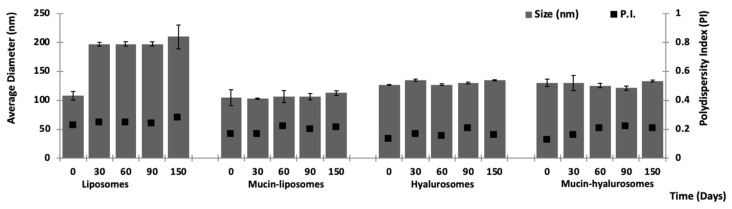
Average diameter and polydispersity index of vesicles stored for 5 months at room temperature (~25 °C). Mean values ± standard deviations are reported (*n* = 3).

The stability of the vesicle dispersions was evaluated by monitoring size, polydispersity index and zeta potential during five months of storage at 25 °C (Figure 2). At 30 days of storage, the liposomes underwent an increase in size, which was doubled. Afterwards, the parameters remained constant. The values of other samples remained constant during the storage. The surface charge of all formulations did not undergo significant variations during the storage (data not shown).

### 3.3. Nebulization Study and Aerodynamic Behavior

The ability of vesicles to improve the aerodynamic properties and accumulation of beclomethasone in the deeper airways was tested using the water dispersion of the drug as reference (Table 3). Using the beclomethasone dipropionate in dispersion, the amount of the drug effectively nebulized in terms of total mass output was ~55%. The same value was reached using liposomes (~59%, *p* > 0.05 versus the value of dispersion). On the other hand, the drug was almost completely nebulized by using the other vesicles containing the polymers (i.e., mucin-liposomes, hyalurosomes and mucin-hyalurosomes) irrespective of their composition. Indeed, the total mass output was ~94% (*p* > 0.05 among the values provided by the three samples).

To better evaluate the behavior of the vesicles under the nebulization process, two other parameters were evaluated: the fine particle dose and the fine particle fraction, which represent the amount and the percentage of beclomethasone dipropionate detected in the last four stages of the impactor. The drug dispersion and drug-loaded liposomes provided a low fine particle dose ~201 μg and fine particle fraction ~38% (*p* > 0.05 between the two values), indicating that only a small part of beclomethasone was effectively nebulized and reached the deeper tract of the respiratory tree. The other vesicles modified and improved with the polymers achieved higher values (fine particle dose ~670 μg and fine particle fraction values ~89% (*p* > 0.05 among values provided by the vesicles containing the polymers). These values are predictive of a good deposition of beclomethasone in the deeper parts of the respiratory tree.

According to these results, the aerodynamic diameter of drug dispersion was the highest (~6.5 µm) followed by that of drug-loaded liposomes (~ 4.86 µm), while that of the other vesicles modified with the polymers was ~3.58 µm irrespective of their composition (*p* > 0.05 among values provided by the containing the polymers). Moreover, it is important to underline that for these last samples, the aerodynamic diameter was lower than 5 µm, which was within the dimensional range of respirable particles, confirming the good potential of the vesicles to be nebulized in the deeper airways.

### 3.4. Effect of Formulations on Viability of Bronchial Epithelial Cells

**Figure 3:**
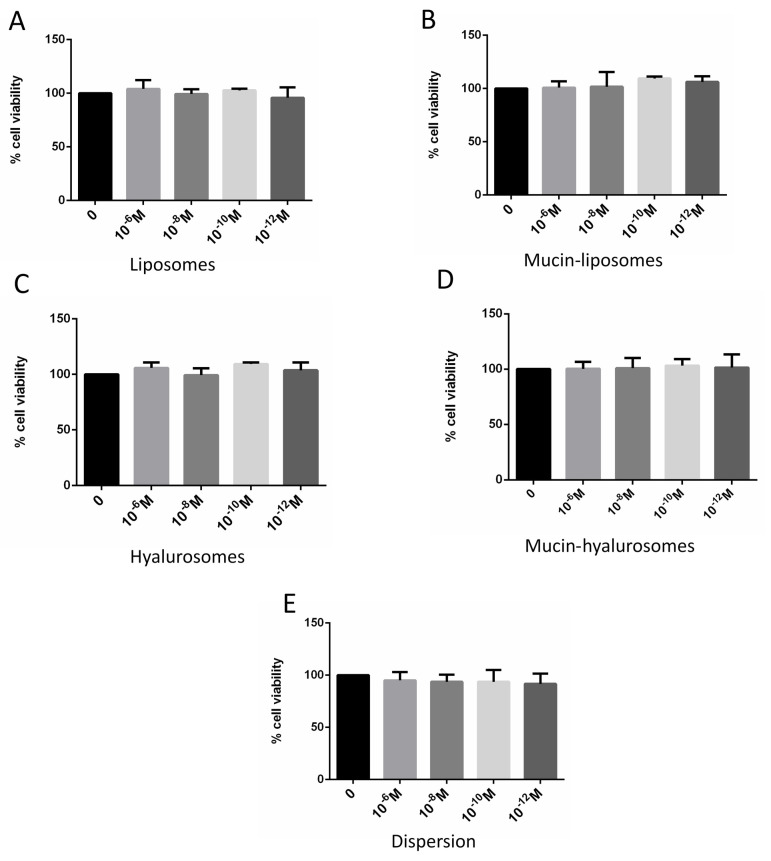
Cell viability of 16HBE incubated for 24 h with beclomethasone dipropionate in dispersion (**E**) or loaded in liposomes (**A**), Mucin-liposomes (**B**), Hyalurosomes (**C**) and Mucin-hyalurosomes (**D**). Mean values ± standard deviations (error bars) are reported (*n* = 6).

The biocompatibility of beclomethasone dipropionate loaded vesicles were tested in vitro by using 16HBE bronchial epithelial cells, whose metabolic activity was evaluated by means of the MTS viability assay. These cells were chosen as a model because they represent the first pulmonary barrier against environmental and pollutant inhaled substances as well as the first target of inhaled drugs. The cells were treated for 24 h with beclomethasone dipropionate in dispersion or loaded in vesicles (Figure 3). The free beclomethasone in dispersion was highly biocompatible, as the resulting cell viability was always ≥90%, irrespective of the concentration tested. The same behavior was obtained using drug-loaded vesicles, confirming their nontoxicity and, thus, their suitability for further studies.

### 3.5. Ability of Vesicles to Counteract the ROS Expression Induced in Bronchial Epithelial Cells

**Figure 4:**
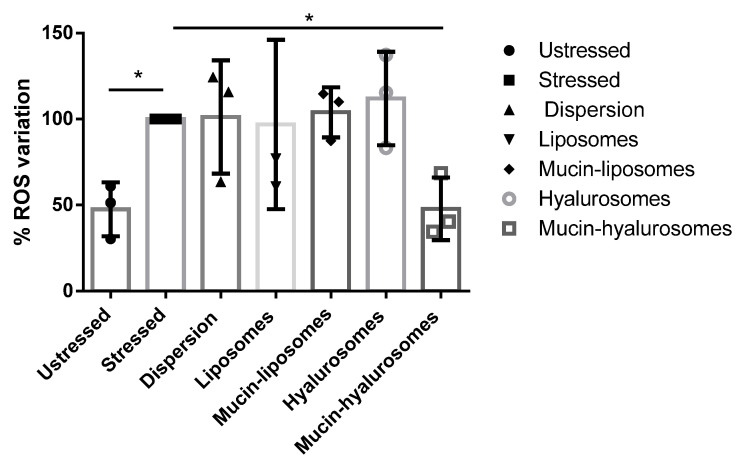
ROS expression in unstressed cells or cells stressed with cigarette smoke extract (20%) and treated with beclomethasone dipropionate in dispersion or loaded in vesicles (10^−9^ M). Mean values ± standard deviations (error bars) are reported (*n* = 3). Symbol (*) indicates values statistically different (*p* < 0.05) to that of stressed and untreated cells.

Previous studies have demonstrated that corticosteroids in dispersion or in solution are not capable of counteracting the increased production of ROS caused by cigarette smoke extract exposure in bronchial epithelial cells, but its loading in phospholipid vesicles seemed to be a promising strategy [28]. The efficacy of the different formulations in inhibiting the production of ROS induced by stressing bronchial epithelial cells with cigarette smoke extract has been assessed (Figure 4). Beclomethasone dipropionate was used at a concentration of 10^−9^ M because it was more effective on the basis of preliminary dose–response experiments (data not shown) and of a previous study [33]. The bronchial epithelial cells unstressed with cigarette smoke extract expressed a basal value of ROS ~47%, due the presence of endogenous species. The exposure of the cells to the cigarette smoke extract significantly increased ROS expression up to ~100% (*p* < 0.0003 versus unstressed cells). Beclomethasone dipropionate in dispersion or loaded in liposomes, mucin-liposomes or hyalurosomes was not able to counteract the ROS production caused by the cigarette smoke extract on epithelial bronchial cells. Indeed, the values were still ~100% (*p* > 0.05 versus ROS positive cells, stressed and untreated). However, beclomethasone dipropionate loaded mucin-hyalurosomes significantly reduced the ROS expression induced by cigarette smoke extract (*p* < 0.01 versus ROS positive cells, stressed and unstressed) reaching the same ROS value as the unstressed heath cells.

## 4. Discussion

The lung administration of drugs has aroused considerable interest in the last years, especially because of the rapid onset of action, the reduction of secondary effects, the removal of the need for professional care, and improved patient compliance [34,35]. Moreover, the recent advancements in nanotechnological carriers have improved the therapeutic achievements of the payload, facilitating its specific delivery to the desired tissues or cells [36]. Considering the low adverse effects addressed by aerosol therapy with nanocarriers, it should be also proposed as a smart preventive treatment to counteract the damaging effects of cigarette smoke in the lungs [36]. Beclomethasone dipropionate is a good anti-inflammatory candidate, which can prevent and treat these damages, especially in smokers, widely affected by asthma or chronic obstructive pulmonary disease [37,38]. Previous studies confirmed that the severe adverse effects of this drug are linked to systemic administration and are highly reduced by inhalation route [39]. Glucocorticoids like beclomethasone dipropionate are widely used and commercialized as suspension or solution. However, its delivery in ad hoc formulated phospholipid vesicles can improve its residence time in the lungs and its internalization inside the cells, thus potentiating its effectiveness. According to our previous studies, in the present work, phospholipid vesicles were selected as carriers for the lung nebulization of beclomethasone dipropionate [40]. Alternatively to liposomes, hyalurosomes were used considering their optimal stability and resistance to mechanical stress. Both formulations were further improved with mucin, which is a multifunctional glycosylated protein increasingly used in biomaterials thanks to its muco-adhesiveness and ability to modulate the immune response [41]. In addition, previous studies disclosed the capability of mucin to provide significant protection against oxidants [4,41]. The main physicochemical characteristics of prepared vesicles were very similar and the addition of hyaluronan only allowed a small increase in the mean diameter with respect to that of the liposomes, probably due to its distribution on the external surface of the vesicles [42]. The addition of mucin did not affect these characteristics. On the contrary, the addition of both hyaluronan and mucin ameliorated the aerodynamic properties of the dispersions [43]. Indeed, the total mass output, the fine particle dose and the fine particle fraction of the vesicular dispersions containing the polymers were higher than those of liposomes, while the aerodynamic diameter was lower than 5 µm, which was within the dimensional range of respirable particles [44]. The results confirmed the better ability of the used vesicles to be nebulized in comparison with the drug dispersion, which is actually used in clinical therapy. The vesicles promoted the delivery of beclomethasone in the deeper airways, where the protection and the treatment of the tissues damaged by cigarette smoke are needed [38]. The entrapment of beclomethasone into the vesicles significantly improved its nebulization, as beclomethasone-loaded vesicles reached the deeper airways to a better extent than the drug dispersion. Moreover, the better aerodynamic properties of the vesicles prepared with hyaluronan and mucin should be related to the higher stability of these vesicles, which were not broken during the aerosolization process. The last is a very strong process involving repeated cycles in which formulation droplets are formed and a fraction of them are sprayed while the rest fall and restart the cycle [45]. During this process, considerable forces are applied, which are responsible for the fragmentation of some vesicles and the loosening of the drug and the new aggregation of empty vesicles [14,25]. These phenomena can address the slightest change in the structure or surface properties of the aerosolized droplets because the drug remains in the water dispersion, and its final fate is like that contained in the drug dispersion, while its loading in vesicles is necessary to achieve a droplet size in the respirable range and a maximum shelf life [43].

One of the essential requirements for the use of new drug delivery systems at lung level is that they must be highly biocompatible at cellular level [46]. The biocompatibility of beclomethasone dipropionate-loaded vesicles against bronchial epithelial cells was around 100%, irrespective of the used concentrations and components, as previously reported for phospholipid vesicles [47]. The efficacy of the formulations was evaluated using beclomethasone dipropionate at a concentration of 10^−9^ M, because, on preliminary dose–response experiments and in a previous study, at this concentration it was more effective [33]. The loading of beclomethasone in the phospholipid vesicles and the composition of the vesicles are key parameters capable of inhibiting the ROS production induced by cigarette smoke. Indeed, previous studies have demonstrated that corticosteroids in dispersion or in solution are not capable of counteracting the increased production of ROS caused by cigarette smoke extract exposure in bronchial epithelial cells [28]. Liposomes, mucin-liposomes and hyalurosomes did not reduce the ROS production induced by cigarette smoke extract, while mucin-hyalurosomes were able to counteract the effect of cigarette smoke and reported the ROS value up to that of unstressed cells. Thus, the combination of mucin and hyaluronan imparts optimal properties to the phospholipid vesicles. Hyaluronan can improve the adhesion of vesicles to the bronchial epithelial cells by biding the CD44, a cell surface proteoglycan involved in cell–cell adhesion, cell–matrix interactions, and lymphocyte activation [48]. It is abundant in many tissues, such as bronchial epithelium [49]. In addition, it is overexpressed in damaged areas of the bronchial epithelium, especially in asthmatic subjects [50]. Then, we can suppose an improved targeting in smokers with compromised airway tissue. The addition of hyaluronan alone to liposomes loading beclomethasone did not ensure a significative reduction in ROS production while the simultaneous presence of mucin potentiated the performance of the vesicles, significantly reducing the ROS production. This effect should be related to the capability of the mucin to provide significant protection against oxidants [4,41,51]. Indeed, the mucin naturally occurring in the mucus exert a scavenger activity against the ROS [52]. Thus, the combination of beclomethasone diproprionate, mucin and hyaluronan imparts optimal properties to phospholipid vesicles, giving them the ability to effectively counteract oxidative damages.

## 5. Conclusions

The addition of hyaluronan or hyaluronan and mucin to phospholipid vesicles allowed us to obtain vesicles which can be easily nebulized, promoting the deposition of beclomethasone dipropionate in the last stages of the impactor, which mimic the lower airways of the respiratory tree. Beclomethasone-loaded vesicles were made biocompatible by using bronchial epithelial cells, but only the mucin-hyalurosomes modified with hyaluronan and mucin effectively counteracted the oxidative stress induced in cells by cigarette smoke, probably due to the targeting effect of hyaluronan and the antioxidative activity of mucin. Overall, the results suggest their possible use as smart treatment to control the damage caused by cigarette smoke.

## Figures and Tables

**Table 1:** Amount (mg/mL) of components used to prepare beclomethasone containing vesicles.

	S75 (mg/mL)	Beclomethasone Dipropionate (mg/mL)	Sodium Hyaluronate (mg/mL)	Mucin (mg/mL)
**Liposomes**	60	1	5	--
**Mucin-liposomes**	60	1	5	0.5
**Hyalurosomes**	60	1	5	--
**Mucin-hyalurosomes**	60	1	5	0.5

**Table 2:** Average diameter (AD), polydispersity index (PI), surface zeta potential (ZP) and entrapment efficiency (E) of beclomethasone dipropionate loaded vesicles. Mean values ± standard deviations are reported (*n* = 6).

	AD (nm)	PI	ZP (mV)	E%
**Liposomes**	108 ± 7	0.22	−9 ± 3	84 ± 2
**Mucin-liposomes**	104 ± 14	0.16	−12 ± 1	88 ± 6
**Hyalurosomes**	127 ± 1	0.12	−12 ± 1	81 ± 6
**Mucin-hyalurosomes**	130 ± 6	0.12	−11 ± 2	86 ± 5

**Table 3:** Total mass output (TMO), fine particle dose (FPD), fine particle fraction (FPF) and aerodynamic diameter (MMAD) of beclomethasone dipropionate in dispersion or loaded into vesicles. Average values ± standard deviations are reported (*n* = 4).

	TMO (%)	FPD (μg)	FPF (%)	MMAD ± GSD
**Dispersion**	55 ± 2	180 ± 3	36 ± 5	6.36 ± 1.15
**Liposomes**	59 ± 4	222 ± 6	40 ± 3	4.86 ± 1.20
**Mucin-liposomes**	94 ± 6	760 ± 2	91 ± 3	3.69 ± 1.28
**Hyalurosomes**	95 ± 3	578 ± 9	87 ± 7	3.50 ± 1.30
**Mucin-hyalurosomes**	93 ± 8	673 ± 4	88 ± 5	3.56 ± 1.30
